# Experimental Advances in the Real-Time Recording of Cross-Linking Alginate *In Situ* Gelation: A Review

**DOI:** 10.3390/polym15132875

**Published:** 2023-06-29

**Authors:** Ioanna N. Besiri, Thomas B. Goudoulas, Ehsan Fattahi, Thomas Becker

**Affiliations:** Research Group of Fluid Dynamics, Chair of Brewing and Beverage Technology, TUM School of Life Sciences, Technical University of Munich, Weihenstephaner Steig 20, 85354 Freising, Germany; ioanna.besiri@tum.de (I.N.B.); ehsan.fattahi@tum.de (E.F.); tb@tum.de (T.B.)

**Keywords:** alginate, cross-linker, *in situ* gelation, hydrogels, beads, kinetics, reaction, diffusion, rheology, structural characterization

## Abstract

Alginate-based hydrogels are promising smart materials widely employed in the food, bioengineering, and energy sectors. The development and optimization of their production require a thorough knowledge of gelation. In recent years, advanced experimental procedures have been developed for real-time cross-linking alginate reaction monitoring. Novel methods, such as customized rheometric setups, enable the recording of mechanical properties and morphological changes during hydrogel formation. These innovative techniques provide important insights into the gelation stages, the reaction rate, the diffusion of cross-linker to polymer chains, and the homogeneity of the gelling structures. Based on real-time experimental data, kinetic models are developed to enhance comprehension of the reaction mechanism and, eventually, to predict the gelation progress. The aim is to enable better control of the characterization of both the complex gelation and the propagated structures. This review aspires to present a comprehensive overview and evaluation of the breakthrough innovations of the real-time *in situ* recording of cross-linking alginate hydrogels and bead formation. A detailed analysis of the pioneering experimental developments provides a deep comprehension of the alginate gelation, including the parameters controlling the reaction.

## 1. Introduction

**Figure 1:**
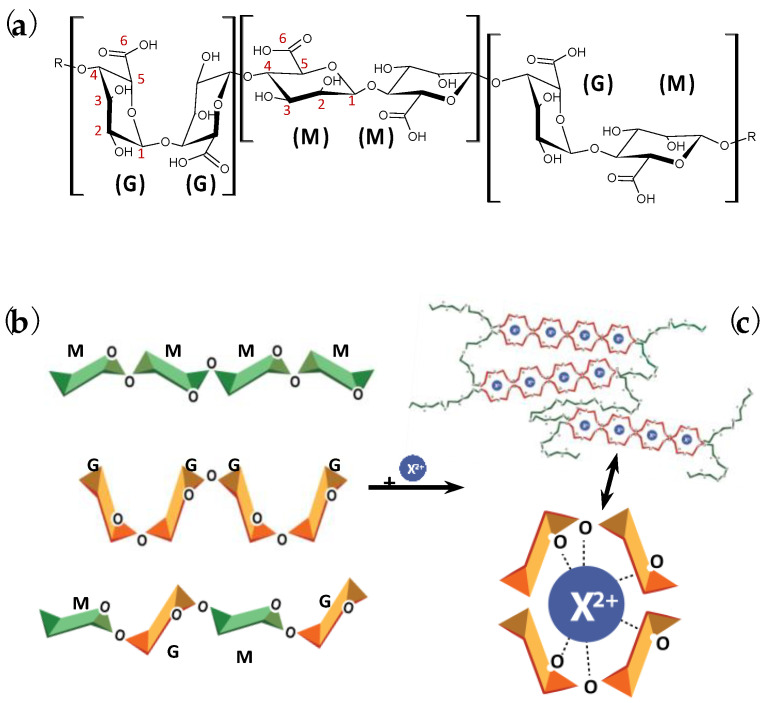
(**a**) Schematic representation of mannuronic (M) and guluronic (G) acid monomers. (**b**) Graphical illustration of alginic acid consisting of MM-, GG-, and MG- sequences. (**c**) The reaction of cations (X^2+^) with alginate chains results in the formation of ‘egg-box′ structures [38]. Reproduced with permission from ACS, Urbanova et al. (2019), *Biomacromolecules*, 20, 11, 4158–4170 (https://pubs.acs.org/doi/10.1021/acs.biomac.9b01052). Further permissions inquiries related to the material excerpted should be directed to the ACS.

**Figure 2:**
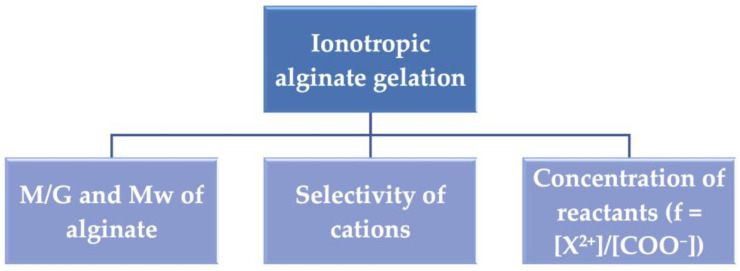
Parameters determining the ionotropic alginate gelation.

The favorable characteristics of alginate, such as biocompatibility, biodegradability, and antimicrobial properties [1,2,3], render it an ideal material for developing ‘smart gelling’ products with a wide range of applications in bioengineering, food, and energy domains [4,5,6]. The polysaccharide is primarily extracted from the cell walls of brown seaweeds [7,8]. To easily tailor the structure and characteristics of the macromolecule, biological production by two genera of soil bacteria, i.e., *Azotobacter* and *Pseudomonas*, is feasible as well [9,10]. Alginate is composed of (1–4)-linked β-D-mannuronic (M) and α-L-guluronic (G) acid monomers arranged in different sequences, i.e., MM-, GG-, and MG-, along the negatively charged long linear chain, as shown in Figure 1a [11,12]. Gelling networks are formed in the presence of alginate in aqueous solutions [13,14]. At a pH lower than the pKa of uronic acid, hydrogels are formed, while the reaction of the polysaccharide with divalent cations results in well-known ionic hydrogels [15,16]. The ionotropic gelation is based on the ‘egg-box′ model, presented in Figure 1b,c, which arises from the initial chelation of cations with the alginate chains and the following interchain association, [17]. It critically depends on the physicochemical characteristics of the reactants, such as their concentration, the kind of cations, and the molecular weight, Mw, and M/G of alginate, as shown in Figure 2 [18]. The divalent metals bind mainly with G- and MG- sequences of the polymer backbone through physical cross-linking [19,20,21]. The dimerization of alginate chains is induced, and multimer structures are formed following the zipper mechanism [22,23]. The selectivity of the polysaccharide with cations increases in the order Mg^2+^ < Co^2+^, Ni^2+^, Zn^2+^ < Ca^2+^ < Sr^2+^ < Ba^2+^ < Cd^2+^ < Cu^2+^ < Pb^2+^ [24,25]. The reaction is controlled by the stoichiometric mole ratio f = [X^2+^]/[COO^−^], where X^2+^ represents the divalent cations. As f increases, the gelation is faster, and stiffer structures are formed [26,27]. Moreover, the alginate gelation can originate from chemical cross-linking. For instance, some bivalent cations and trivalent metals, as well as other chemical intermediates such as epichlorohydrin, cross-link with alginate chains through strong covalent bonds [20,21]. Additionally, to fabricate materials with enhanced mechanical strength, alginate can create double network (DN) hydrogels with other macromolecules, such as gelatin or polyacrylamide [28,29].

The breakthrough applications of hydrogels dictate an extensive comprehension of the formation mechanism [30,31]. Advanced methods, such as rheology and light-triggered techniques, allow for recording of the gelation kinetics *in situ* [32,33]. Monitoring the process determines the stages of the reaction, the factors which evolve in each stage, and the temporal micro-structural rearrangements [34,35]. Based on real-time experimental data, kinetic models are developed to enable a deeper understanding and accurate prediction of the gelation progress. This leads to the development of strategies to optimize the characterization of complex gelation [36,37]. The present review demonstrates and evaluates the advanced *in situ* methods for monitoring the alginate cross-linking gelation. Novel concepts based on rheology and time-resolved techniques, such as small angle X-ray scattering (SAXS), monitor the alginate hydrogel and bead composition. Information is provided on the temporal evolution of the mechanical properties of the system to detect the dominant parameters affecting the reaction and further elaborate the diffusion mechanism of the cross-linker to alginate solution. Additionally, the visualization of gelation captures the changes in the gelling structures and investigates their homogeneity. With a thorough assessment of the *in situ* methods, the challenges and opportunities of real-time gelation recording are discussed to shed light on potential future research that could further advance this domain.

## 2. Introduction to *In Situ* Gelation Methods

**Figure 3:**
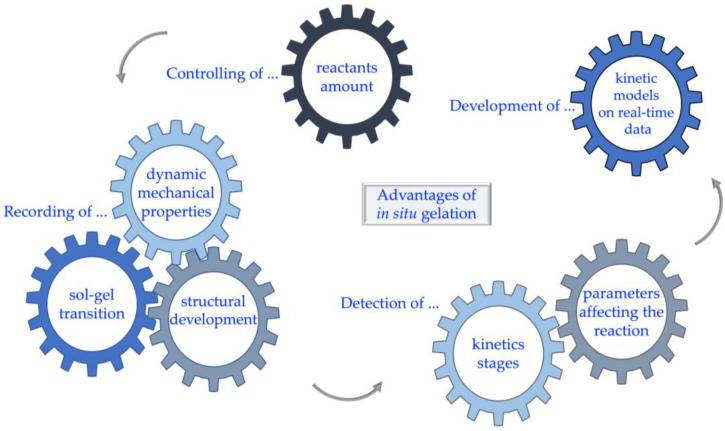
Advantages of *in situ* gelation.

The innovative applications of polymers require detailed knowledge of their molecular characteristics and reaction mechanisms [39,40]. The conventional ex situ preparation of hydrogels limits their study to a steady-state of kinetics, impeding a comprehensive investigation of their dynamic structural and mechanical properties [41]. In contrast, the *in situ* methods provide an opportunity to record the gelation in its original position from the initiation of the reaction [42,43]. As Figure 3 shows, *in situ* gelation can facilitate the precise control of the volume and concentration of reactants to modulate the reaction accurately. Furthermore, the detection of gel point, i.e., the instant that the transition from solution to gel phase occurs [44], is achieved along with the recording of the dynamic mechanical response and structural development of hydrogels. In this way, the kinetic stages and the parameters defining each reaction step are determined. Finally, kinetic models can be developed based on real-time experimental data to describe and predict the reaction.

Table 1, which is at the end of the review, includes the methods developed during the last years to characterize the cross-linking alginate *in situ* gelation. In brief, the mechanical properties of alginates are controlled by advanced rheometric and light-triggered techniques, as well as by cantilever sensors. Moreover, innovative procedures based on time-resolved methods, such as small-angle Χ-ray scattering, Fourier-transform infrared spectroscopy (FTIR), and dark field microscopy (DFM), are designed to detect the microstructural and macrostructural rearrangements during the gelation. A detailed description of these techniques is presented in Section 3 and Section 4.

*In situ* experimental procedures are used to form alginate hydrogels or alginate beads. The hydrogels are generated by the diffusion of the cross-linking agent into alginate solution [45], while the beads derive from the drop-wise methods in which alginate solution drops into pools of gelling solution [46]. The source of the cross-linker determines whether the gelation is internal or external [47]. In the first case, insoluble salt is added to the alginate solution, and the cations diffuse to the chains of the polymer when the appropriate pH conditions are achieved by a catalyst such as D-glucono-δ-lactone (GDL). The slow hydrolysis of GDL releases the protons, and they react with the biopolymer [48,49]. In the second case, the gelation is achieved by the direct exposure of alginate solution to an active form of cations, specifically, a soluble salt [50,51]. The external gelation implies a faster reaction than the internal mechanism, but the slower internal gelation leads to more homogeneous gelling structures [52,53].

## 3. *In Situ* Real-Time Recording of Alginate Hydrogel Formation

### 3.1. Conventional Rheometric Setups

Real-time recording of gelation kinetics provides valuable insights into the cross-linking reactions of alginate during hydrogel formation. Rheology, the study investigating the flow of matter [54], is extensively used to evaluate the progress of gelation in dynamically developing systems [55,56]. Specifically, oscillatory time sweeps are performed to study the temporal evolution of the mechanical properties of materials undergoing time-dependent structural rearrangements [57]. The mechanical properties are represented by the storage (G′) and loss (G″) moduli, showing the elastic and the viscous responses of the system, respectively. When G″ > G′, the material behaves as a solution, while when G″ < G′, the gel behavior predominates [54,55,57]. At the crossover point of the moduli, the first microgel structures are created and the reaction initiates [54]. For this reason, it can be called the gel point, which is accurately determined by *in situ* rheological experiments, leading to the detection of the precise gelation time, in contrast to the vial inversion test, which detects gelation time by inverting the vials until the material no longer flows [58,59]. Over the years, the most common method for conducting these dynamic experiments has been the utilization of conventional rheometric plates [34,35,60]. Typically, a uniform mixture of reactants is prepared ex situ, and it is then applied to the bottom plate of the rheometer directly after mixing, where it is subjected to dynamic oscillatory tests [61,62]. The recording of modulus kinetics is effective and successful [63,64]. This rheological procedure is mainly applied to study internal gelation, as the slow reaction provides enough time for sample placement and initiation of the oscillatory experiments within the solution state of the material [65].

The study of Funami et al. [66] is remarkable, utilizing internal gelation to investigate the kinetics under different physicochemical characteristics of reactants. Alginate hydrogels rich in G-residues are more elastic in the presence of higher CaCO_3_ concentrations (15 mM), while the elasticity increases in those rich in M-residues in the lowest availability of cations (3.75 mM). They obtain rod-like and network-like conformations, respectively, as shown in the additional atomic force microscopy images. Based on long-term oscillatory time sweeps, the authors propose the following first-order kinetic equation to describe the temporal evolution of the storage modulus, G′:

(1) $$G^{\prime }\left(t\right)=G_{sat}^{\prime }\left\{1-\exp\left[-k_{t}\left(t-t_{0}\right)\right]\right\},$$

Equation (1) has been applied to determine the gelation time (t_0_), the rate constant (k_t_), and the saturated storage modulus ($G_{\mathrm{sat}}^{\prime }$) of alginate hydrogels, i.e., the equilibrium modulus as t reaches infinity [66,67]. In their following research work, a simpler equation with one exponential factor was employed to describe the internal alginate gelation adequately [67].

Similarly, Fernández Farrés et al. [68] have investigated the *in situ* formation of alginate-Ca^2+^ fluid hydrogels using a four bladed vane in cup on a Kinexus Rheometer to control the shear profile. During the reaction, the viscosity increased due to the propagation of ‘egg-box′ structures, while the shear rate caused a break-up in the network, and micro-gelled particles were formed. Under constant concentrations of reactants, the lower shearing (100 and 200 s^−1^) results in higher viscosities, while the higher ones (400 and 600 s^−1^) may cause a delay in the association of the initially formed particles and decrease the reaction rate. Based on real-time data of G′ obtained by dynamic oscillatory experiments with cone-and-plate geometry directly after the formation of structures at different shear rates, kinetic models are proposed to describe the gelation. An exponential equation applied to the high-Mw alginate hydrogels shows that the kinetic constant is the same for all structures, meaning that, at the time of shear removal, the strength rate is independent of the external forces, since the processing period is higher than the gelation time. For the reaction of low-Mw alginates, a linear first-order model has been employed, revealing that the ordering rate increases when hydrogels are formed at low shear rates, which accelerates the development of particles [68].

**Figure 4:**
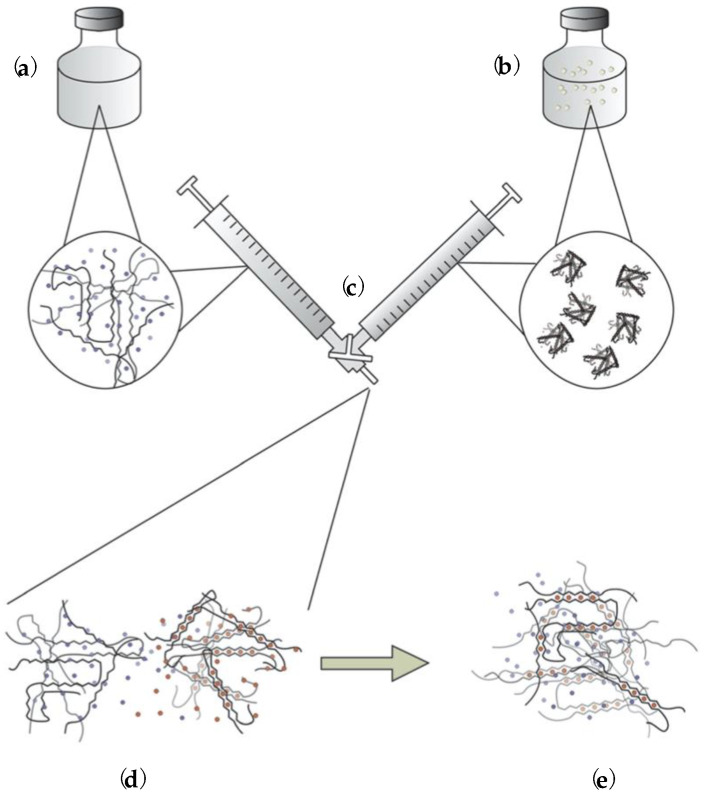
(**a**) Aqueous solution of sodium alginate. (**b**) Aqueous solution consisting of insoluble strontium or calcium alginate particles. (**c**) Mixing of components. (**d**) Gelling ions migrate from strontium or calcium alginate particles to the alginate chains. (**e**) Formation of gel [69].

Another interesting study reports a customized mixing of reactants by developing a syringe-induced technique, one which utilizes internal gelation [69]. In brief, Larsen et al. [69] prepare one syringe containing soluble alginate and one with insoluble alginate/gelling ion particles, as shown in Figure 4a,b. Through a three-way connector, as presented in Figure 4c, the soluble alginate is transferred to the other syringe, and, upon mixing of reactants, it is placed through a channel onto the standard plate of the rheometer to record the gelation which occurs according to the mechanism in Figure 4d,e. The effect of cations on gelation kinetics can be controlled, and the proposed exponential kinetic equation, describing the temporal evolution of G′, shows that the gelling rate is determined by the release rate of cations from the particles [69].

Furthermore, the conventional rheometric setups serve to study the temperature control kinetics of cross-linking alginate gelation due to the attached Peltier system [70,71]. Goudoulas et al. [72] have successfully studied the phase-transition kinetics of binary alginate–gelatin mixtures by applying isothermal time sweeps in a temperature range (5–20 °C) using a parallel-plate configuration on an Anton Paar MCR 502 rheometer. Additionally, the standard rheometric configurations can record the reaction of alginate with a covalent cross-linking agent upon ex situ mixing of reactants, since the sol–gel transition occurs relatively slowly. This has been achieved by Li et al. [73] in order to study the reaction of N,O-carboxymethyl chitosan with oxidized alginate without additional cross-linkers, as well as by Yang et al. [74] to record the gelation of alginate with adipic acid dihydrazide through coupling agents. However, they cannot provide adequate monitoring of the external gelation, which occurs instantaneously, i.e., within the first seconds of mixing the reactants.

### 3.2. Advanced Rheometric Setups

Advanced rheometric methods have been designed to enhance the investigation of the mechanical response in diffusion-controlled alginate hydrogels [75]; for instance, when crafted polysaccharide is exposed to light, conformational changes occur, resulting in gelling networks [76]; Bonino et al. [77] have utilized the light emitting diode (LED) bottom plate of a TA stress-controlled rheometer to monitor the gelation of alginate chemically modified with methacrylate groups when a photoinitiator presents in the system. This method facilitates the detection of the gel point and the evaluation of the reaction kinetics. Higher UV intensities accelerate the sol–gel transition and increase the storage (G′) and loss (G″) moduli at a constant concentration of the system. Additionally, based on short-term UV oscillatory experiments, an empirical exponential model is applied tο obtain the moduli at steady-state, which is found to be critically dependent on the degree of methacrylation [77]. Subsequently, the same research group observed that in addition to cross-linking due to the light-induced method, there is evidence of micro-structural development in the absence of UV light, i.e., dark curing. This is attributed to the acidic pH of the mixture, which facilitates the CaCO_3_ dissociation and the cross-linking with alginate chains. Subjected or not to dark curing, the final properties of gelling formations are unaffected, reaching the same modulus plateau, but the gelation rate slows down when UV is not continuously applied to the gelation [78].

**Figure 5:**
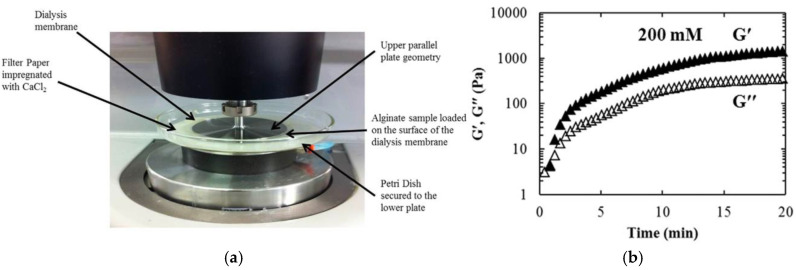
(**a**) Modification of a lower rheometric plate on Malvern Gemini Nano HR rheometer. (**b**) Temporal evolution of G′ (open symbols) and G″ (filled symbols) of alginate–Ca^2+^ reaction [79]. Reprinted from *Food Hydrocolloids*, 55, M.H. Mahdi, R. Diryak, V. Kontogiorgos, G.A. Morris, A.M. Smith, “*In situ* rheological measurements of the external gelation of alginate”, 77–80, Copyright (2016), with permission from Elsevier.

The external cationic alginate gelation exhibits rapid kinetics and forms heterogeneous structures, facts making it challenging to *in situ* monitor the mechanical properties using the conventional rheometric plates. To address this issue, innovative customized rheometric setups have been developed. In 2016, Mahdi et al. [79] demonstrated a modified lower rheometric plate, as presented in Figure 5a, facilitating the initiation of reaction at the instrument. It consists of a petri dish with filter paper impregnated with CaCl_2_ solution and a hydrated dialysis membrane on the top. The alginate solution is placed there, preventing its absorption by the filter paper. The fast initiation and the subsequent slower evolvement of the reaction can effectively be recorded, as shown in Figure 5b. Additionally, the systematic increase of moduli in the presence of more cations in the system can be recorded. Interestingly, the design enables the monitoring of hydrogel degradation by replacing the CaCl_2_ filter with one containing a calcium chelator [79], while it has been utilized to study the effect of the physiological fluids on the mechanical response of cross-linking hydrogels [80]. Based on these advantages, a rheo-dissolution cell has been developed controlling both the *in situ* gelation and the drug release profile of gelling systems [81].

**Figure 6:**
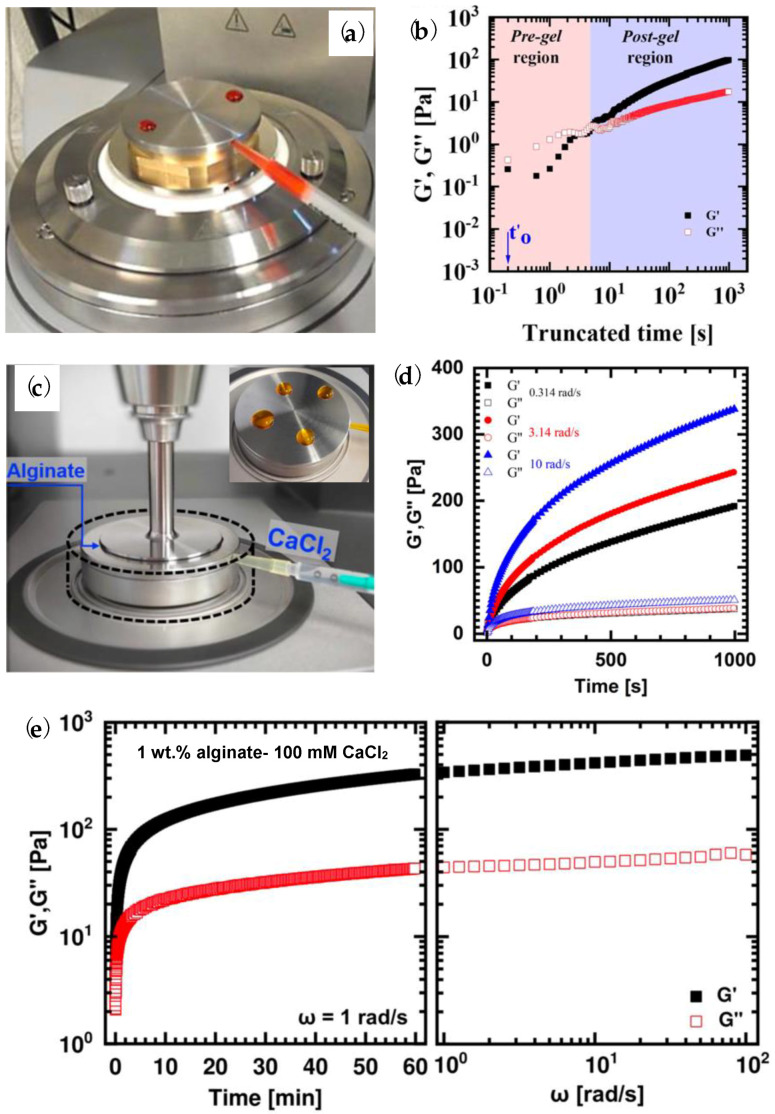
(**a**) Customized rheometric setup for cross-linking reactions on an Anton Paar MCR 502 rheometer. (**b**) Detection of the sol–gel transition in an alginate–Ca^2+^ reaction [82]. Reprinted from *Carbohydrate Polymers*, 246, Besiri, I.N.; Goudoulas, T.B.; Germann, N., “Custom-made rheological setup for real-time fast alginate-Ca^2+^ *in situ* gelation”, 116615, Copyright (2020), with permission from Elsevier. (**c**) Improved setup and final configuration of Kinexus Ultra+ rheometer. (**d**) Kinetics at different angular frequencies when 1 wt.% alginate reacts with 100 mM Ca^2+^, and (**e**) long-term response of alginate-Ca^2+^ gelation, using the four micro-holes configuration [83]. Reprinted from Besiri, I.N.; Goudoulas, T.B.; Germann, N. “Impact of CaCl_2_ concentration and *in situ* rheometric setup configuration on fast alginate–Ca^2+^ reaction”. *Phys. Fluids* **2022**, *34*, 053104, with the permission of AIP Publishing.

**Figure 7:**
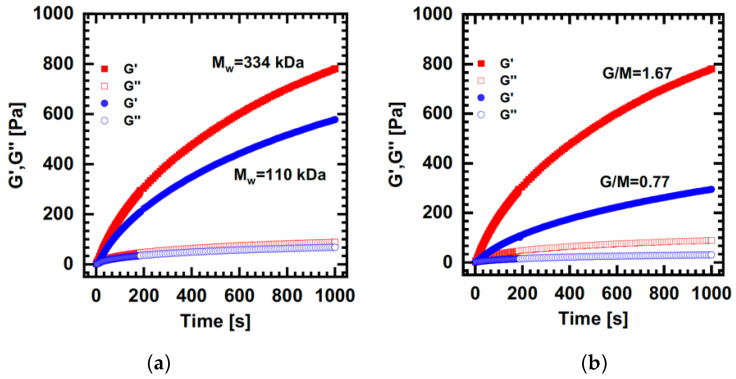
Time sweeps of 1.2 mL 0.5 wt.% alginate—0.6 mL 25 mM CaCl_2_ using the setup of Besiri et al. [83]. Comparison of kinetics using alginates with different (**a**) Mw and (**b**) G/M [84]. Reproduced with permission from Didonaki, A., *Comparison of hydrogels using in situ characterization*. Master’s Thesis; published by Technical University of Munich School of Life Sciences, 2022.

In 2020, Besiri et al. [82] introduced an advanced setup for mixing the reactants on the rheometer during the external gelation, one resembling a batch reactor operation. It consists of two parts with an inner void when coupled together. A cross-linking agent fills the internal space with a volumetric syringe connected to a side-feed hole. At the initiation of the rheological measurement, CaCl_2_ is injected through micro-holes into the alginate solution placed on the top of the setup, and the reaction begins. As shown in Figure 6a,c, the design can be easily adaptable to various rheometers with a flexible arrangement of micro-holes to study the process thoroughly. Figure 6b presents that the setup can successfully record the initial and quasi-steady-state kinetic stages and assists in the determination of the fast sol–gel transition, i.e., the crossover of moduli, where the first gelling structures are created. Additionally, the stoichiometry of reactants can be monitored. Higher concentrations and injected volumes of CaCl_2_ increase the growth rate of G′, signifying that the diffusion of cations controls the gelation. By controlling the angular frequency (ω) it is shown in Figure 6d that higher ω results in a sharper increase of G′, i.e., the formation of stiffer hydrogels, which is attributed to the acceleration of the propagation of multimer structures. The configuration can form typical soft hydrogels after 1 h of reaction, as it is observed in Figure 6e by frequency sweep experiments where the moduli are developed at a slow rate independently of the applied ω [82,83]. Further, *in situ* investigation demonstrates that alginates with higher Mw and M/G increase the elasticity of the network due to the higher availability of free polymer chains for cross-linking, as shown in Figure 7 [84]. Currently, the novel setup is utilized to provide insights into external alginate gelation concerning the spatial distribution of the ions’, while a two-kernel equation, consisting of one exponential and one logarithmic factor, seems to describe and predict the reaction adequately [85].

In addition to the innovative rheometric designs, interfacial shear rheology has been utilized to control the *in situ* generation of alginate foam hydrogels [86]. Ben Djemaa et al. [86] propose a gas-induced cross-linking reaction instead of the conventional internal mechanism for hydrogel formation. Gas steam, supplied with CO_2_ through a flow meter, is humidified and connected to a double-wall ring geometry containing an alginate solution with CaCO_3_ particles. The flow of CO_2_ in the interface of the pre-gelling system triggers the gelation. G′ and G″ increase rapidly within 90 min, since the diffusion of CO_2_ causes the acidification of the interface, providing the suitable pH conditions for the cations’ release to alginate chains. Later, the moduli tend to reach a plateau, possibly meaning that the acidification process and the gel front have been developed in the entire system, i.e., from the interface to the bottom of the trough. The researchers state that the physicochemical characteristics of reactants and the CO_2_/air ratio determine the gelation progress and need further investigation [86].

### 3.3. Alternative Techniques beyond Rheology

Apart from employing customized rheometric techniques to understand alginate gelation, researchers propose to use cantilever sensors, as well. A cantilever sensor consists of a beam anchored at one end and having a free extension into space, and it is made from a thin film with mechanical flexibility. The movement of the beam, along with the combination of piezoelectric elements, enables the detection of the deflection of the cantilever, which results in the measurement of the mechanical response of materials. This experimental approach minimizes the time required for monitoring the dynamic properties, while the gelation can occur under high frequencies [87]. Recently, Haring et al. [88] have evaluated alginate-Ca^2+^ kinetics with a piezoelectric-excited millimeter cantilever (PEMC) sensor [89]. In the implementation, it is immersed in a petri dish containing alginate, in which CaCl_2_ is injected 5 mm from the anchor of the cantilever upon stabilization of the signal. In this way, the resonant frequency is recorded and used to calculate the G′ and G″ of the developed gelling structures through fluid–structure interaction models. The gelation kinetics can be evaluated similarly to oscillatory rheological experiments, but the crossover of moduli is not detected due to the high frequency, i.e., G′ > G″ from the reaction’s initiation [88].

**Figure 8:**
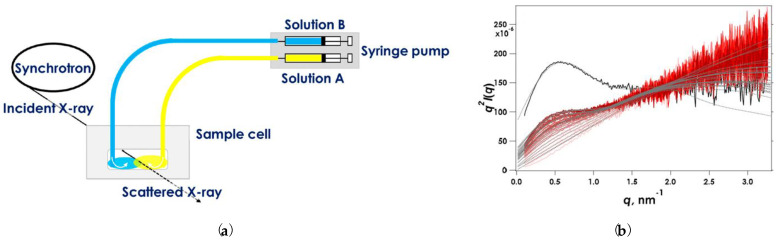
(**a**) Graphical representation of the customized configuration for the time-dependent SAXS on alginate gelation. (**b**) Kratky plots during CLEX gelation of 1% alginate in aqueous MOPS buffer containing CaEDTA and ZnEDDA at Ca^2+^ = 30 mM and pH = 7.0. The data were recorded every 5 s for 145 s. Solid black lines indicate SAXS obtained from fully reacted samples following 10 h reaction time [92]. Reproduced with permission from Yamamoto et al., *Gels*; published by MDPI AG, 2019.

The overall comprehension of the cross-linking gelation requires not only the recording of mechanical properties but also the investigation of the structural conformation [90,91]. Yamamoto et al. [92] have designed a customized sampling system combined with SAXS to *in situ* monitor the polymer chains’ association in alginate–Ca^2+^ structures developed through competitive ligand exchange (CLEX) cross-linking [93,94]. After mixing two alginate solutions, the one containing a cross-linking agent (CaEDTA) and the other an exchange ion (ZnEDDA), Zn^2+^ ions are exchanged with Ca^2+^ ions, which are subsequently available to cross-link with polymer chains. As shown in Figure 8a, syringe pumps injected the solutions in a sample cell so that an interface between them was created, and the reaction was controlled only by the diffusion of reactants. The X-ray irradiated the interface in short time intervals to continuously monitor the process. The Kratky plots presented in Figure 8b show that *q*^2^*I* increases linearly with *q* (I the intensity, and q the magnitude of scattering), meaning that the polymer chain is in a single coil condition at pH = 7, while the maximum value at 0.5 nm^−1^ indicates the chain association in the first 2 min of the experiment. The researchers have observed that, at higher pH, the signal of SAXS is weaker due to higher aggregation and less cross-links of alginate chains. Based on this experimental data, a two-component broken rod model is proposed to evaluate the cross-sectional form of junction zones [92]. Recently, Pragya et al. [95] probed the dynamic structures of an alginate–acrylamide double network in the presence of both cationic and covalent cross-linking agents by applying a series of time-induced methods, such as FTIR, UV-vis and Raman spectroscopy. *In situ* hydrogel characterization after the direct mixing of reactants indicates that the gelation is developed in two phases. Initially, physical entanglements of polymers and covalent cross-linking occur. Later, the cross-linking interactions control the reaction [95].

*In situ* visualization of the process provides important insights into the morphological rearrangements during the reaction [96,97]. Chueh et al. [98] have observed the formation of hydrogels in microchannels upon the release of cations to alginate chains from a caged calcium complex through UV light exposure. The non-gelled alginate is removed from the system through washing, and the gelling structures are dissolved in the presence of EDTA, resulting in the recovery of the flow in channels. The method controls the flow of the system in microfluidic devices applicable to in vitro 3D cell culture. Similarly, Braschler et al. [99,100] have monitored the gel front in an alginate–Ca^2+^ system with a sequential gel layer synthesis in a microfluidic chip, using fluorescent polymer combined with the theoretical study. They reported that the diffusive depletion zone affects the concentration profile on the gel front, while higher flow rates decrease the gel’s growth rate. This may be attributed to an interplay between the more alginate chains diffusing in the gel front, which require more Ca^2+^ ions, and the washing out of partially cross-linked chains during the deposition of layers. Overall, these decrease the concentration of cations in the reaction zone [99,100]. Nunamaker et al. [101] have visualized diffusion-based gelation by dropping CaCl_2_ into an alginate solution consisting of a complex indicator. The gel rate is detected by the color change of the indicator from dark red to yellow in 50% of hydrogel, and it is influenced by the physicochemical properties of reactants, i.e., the higher the cationic concentration, the faster the gel’s development.

**Figure 9:**
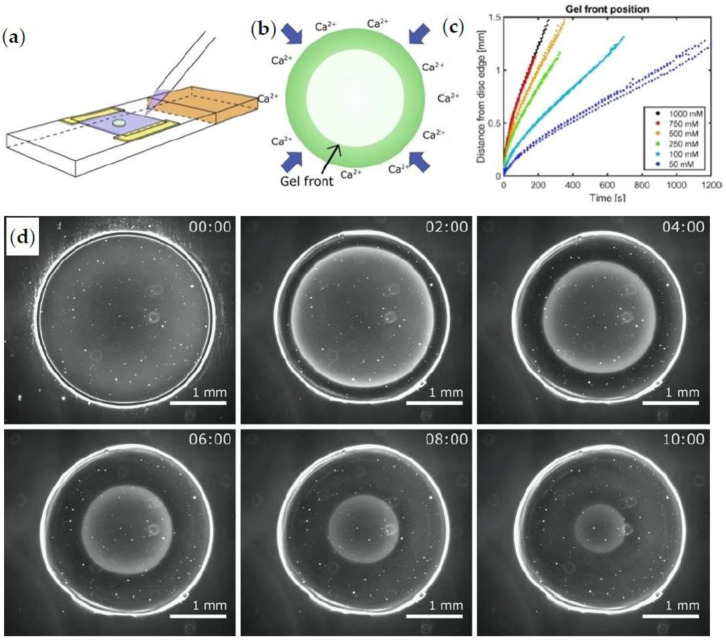
(**a**) Flow cell for the alginate–Ca^2+^ gelation process. (**b**) Graphical representation of the calcium flux (blue arrows) in the gelled alginate (solid green color) (**c**) Temporal evolution of gel front as a function of the CaCl_2_ concentration. (**d**) Dark field (DF) microscopy images show the gel front position at different time intervals within the 100 mM CaCl_2_ flow in the system [104]. Reprinted from *Acta Biomaterialia*, 44, S.H. Bjørnøy, S. Mandaric, D.C. Bassett, A.K.O. Åslund, S. Ucar, J.-P. Andreassen, B.L. Strand, P. Sikorski, “Gelling kinetics and *in situ* mineralization of alginate hydrogels: A correlative spatiotemporal characterization toolbox”, 243–253, Copyright (2016); with permission from Elsevier.

Interestingly, Secchi et al. [102] have investigated the microscopic dynamics of cross-linking gelation by applying photo correlation imaging (PCI), a light scattering method, on a gelling system in which CaCl_2_ slowly permeates through a membrane to an alginate solution. Additionally, the speckle motion in PCI controls the local flow field within the sample, revealing that the gel front is regulated by the diffusion of cations and molecules and critically depends on the convection of reactants through the local flow [102]. Another captivating research effort utilizes dark field microscopy (DFM) to record the progress of the gel front during an alginate cross-linking reaction [103,104]. As shown in Figure 9a, two microscope slides with an alginate droplet in the center, in the gap between them, are gently pressed together to create a flow cell. CaCl_2_ is injected in this space at the periphery of the cell, and the diffusion-controlled reaction initiates. The flux of cations occurs in the radial direction, as Figure 9b shows. In this way, the gel front increases from the periphery to the center of alginate droplet during the cross-linking reaction, as can be observed in the DFM images in Figure 9d. The difference between gelled, i.e., narrow bright band, and ungelled alginate is distinct. This visualization is utilized to calculate the velocity of the gel front. Figure 9c shows that the gel front velocity is higher when more cations are available. In this way, the distance of non-gelled alginate solution from the periphery of the cell sharply increases due to the high number of cross-links formed in the reaction zone. Finally, a reaction–diffusion model is applied to describe and predict the dynamic gel front for a variety of geometries in which the gelation is developed [104].

### 3.4. In Situ Alginate Hydrogels in Membranes

The *in situ* control of cross-linking alginate hydrogels in membranes provides important information for their filtration, which is advantageous for industrial and environmental applications [105]. Hassan et al. [106] have obtained the pseudo-first-order kinetics of multimembrane alginate hydrogels in capillary columns at different temperatures. The gelation is developed rapidly in the beginning and at a slow rate across longer times due to the high density of the network of the gelled membrane [106]. Additionally, Sioutopoulos et al. [107] have investigated *in situ* the mechanical properties of fouled alginate segments on reverse osmosis membranes using a stress-controlled rheometer. The researchers reported that the elasticity increases when low concentration of cations flow to the membrane, possibly due to additional strong cross-links of Na^+^ with alginate macromolecule [107]. Epstein et al. [108] have recently proposed an advanced configuration combining micro-rheology with microcopy to control both the mechanical and conformational changes of a foulant layer at a customized alginate–Ca^2+^ membrane cell connected to a pressurized feed vessel. The mechanical properties are calculated by the particle-tracking method. At higher fluxes, compaction of the foulant layer occurs, and stiffening of the material is observed [108].

## 4. *In Situ* Real-Time Recording of Alginate Bead Formation

The broad implementation of alginate beads in environmental and biological applications highlights their significance [109,110]. Electrodispersion reactors and electrospray methods are only a few of the techniques applied to form cross-linking beads [111,112]. The enhanced comprehension of their synthesis makes the *in situ* control of their reaction kinetics imperative in order to evaluate their dynamic properties [113,114,115]. Lee et al. [116] injected an alginate droplet into baths of calcium sources with mixing times up to 4000 s. An osmotic gradient is created between the beads and the solution bath, which is responsible for cation diffusion to the gelling matrix. By rheological investigation of beads of 8 mm diameter, it was observed that, after 100 s of mixing, the mechanical properties of alginate–CaCl_2_ beads were constant, meaning that the preparation time is short compared to this of calcium lactate and calcium gluconate sources [116]. Additionally, a microfluidic flow-focusing device was employed to produce alginate microparticles, which were then injected into a CaCl_2_ tank. When continuous stirring was applied, the diffusion of cations to alginate particles was accelerated, and their faster solidification was achieved, as opposed to the particles gelled in the non-stirred tank [117].

**Figure 10:**
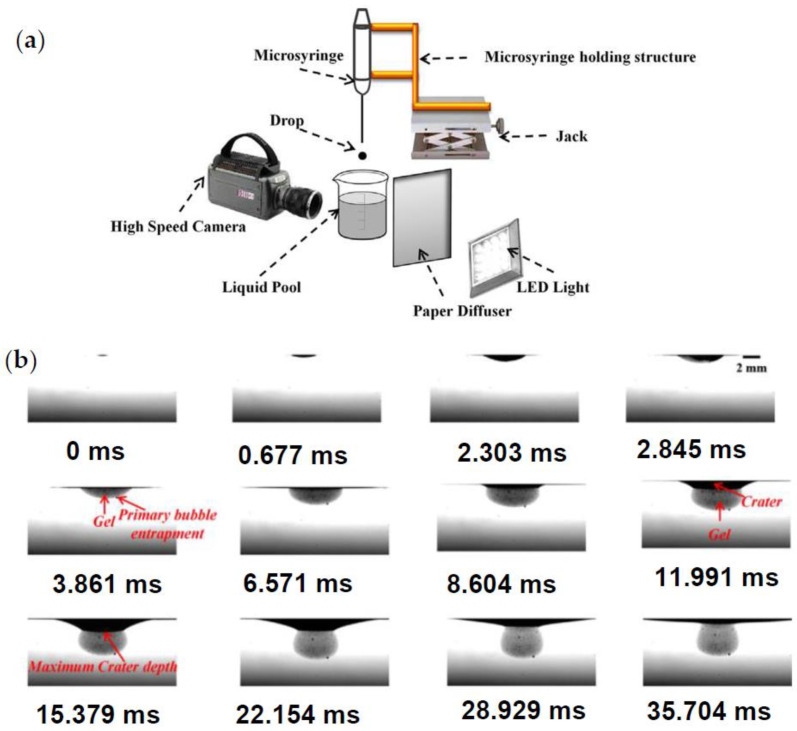
(**a**) Schematic configuration for forming gel beads. (**b**) Successive images of crater dynamics and gel formation for drop impact from 15 mm [120]. Reprinted from *Journal of Colloid and Interface Science*, 528, K. Haldar, S. Chakraborty, “Effect of liquid pool concentration on chemically reactive drop impact gelation process”, 156–165, Copyright (2018), with permission from Elsevier.

Moreover, visualized extrusion-dripping methods are utilized to capture the shape and size of alginate–Ca^2+^ beads, which are affected by the physicochemical characteristics of solutions, the size of the droplets, and the collecting distance [118,119]. Haldar et al. [120,121,122] have developed the configuration presented in Figure 10a. As alginate drops into a calcium solution bath, a high-speed imaging system records the formation of beads. As observed in the sequential snapshots of Figure 10b during gelation, the crater is propagated in the liquid surface of cross-linking agent, while the dynamic structural arrangements are distinct. The crater is restored to its shape upon the injection of the alginate droplet into the CaCl_2_ solution [120].

**Figure 11:**
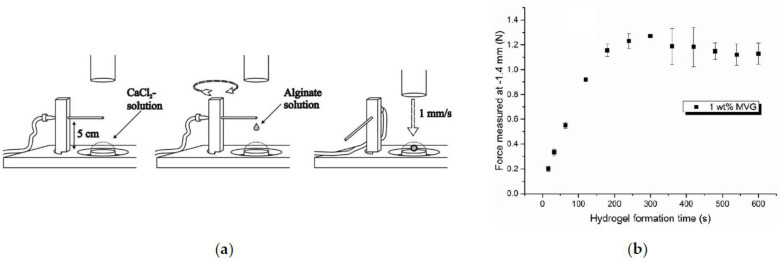
(**a**) Experimental process for forming alginate beads. (**b**) Force of the pressurized beads at 1.4 mm to the sample holder as a function of the hydrogel formation time. The error bars correspond to the standard deviation of three experiments [123]. Reprinted from *Carbohydrate Polymers*, 205, S. Stößlein, I. Grunwald, J. Stelten, A. Hartwig, “In-situ determination of time-dependent alginate-hydrogel formation by mechanical texture analysis”, 287–294, Copyright (2019), with permission from Elsevier.

Furthermore, Stößlein et al. [123] have constructed a customized sample holder on a texture analyzer, as depicted in Figure 11a. The configuration creates inhomogeneous alginate beads with an alginate–Ca^2+^ shell and a core of pure cross-linking agent. Upon the beads’ formation, the beads are pressurized, and their mechanical response is obtained as a function of time, as shown in Figure 11b. At approx. 300 s, a plateau was recorded indicating the two stages of kinetics. The authors also observed a decrease in the volume at 300 s, indicating that the fast gelation will not occur after 300 s [123].

**Figure 12:**
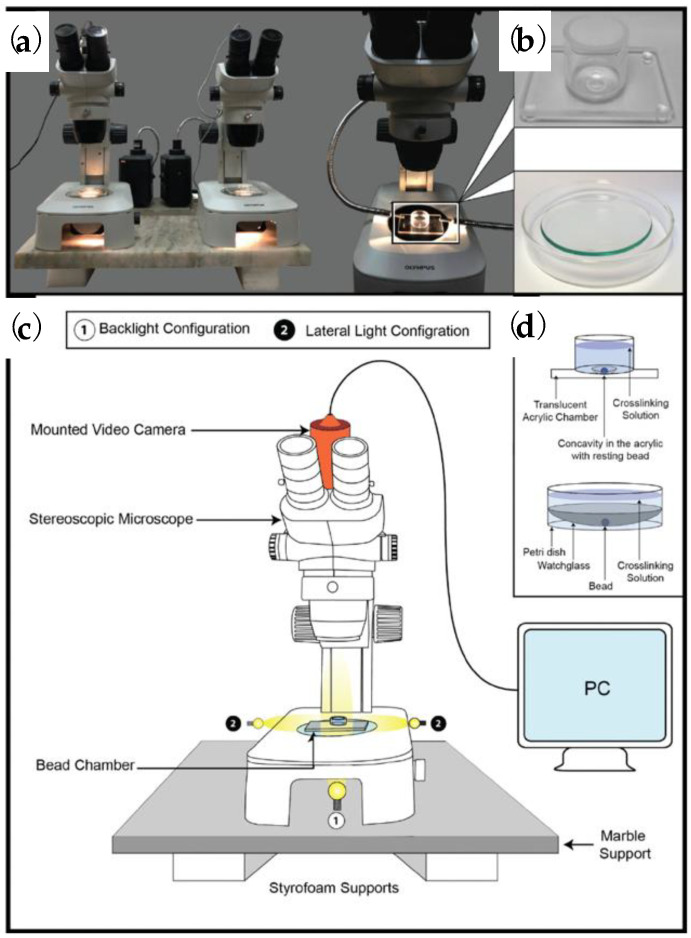
(**a**) Microscopical configuration to measure the temporal bead volume change. (**b**) Two types of chambers are employed in the microscope. (**c**,**d**) Graphical representation describing the setup. [124]. Reprinted from *Carbohydrate Polymers*, 269, A. Posbeyikian, E. Tubert, A. Bacigalupe, M.M. Escobar, P. R. Santagapita, G. Amodeo, M. Perullini, “Evaluation of calcium alginate bead formation kinetics: An integrated analysis through light microscopy, rheology and microstructural SAXS”, 118293, Copyright (2021), with permission from Elsevier.

Recently, Posbeyikian et al. [124] have controlled the gel front in alginate–Ca^2+^ beads using an optical-video method, as presented in Figure 12a–d. The configuration comprises a vibration-stabilized stereoscopic microscope with a digital camera and a specialized chamber to position the bead for undisturbed image focus during cross-linking. Based on this technique, a fast automatic protocol is provided for the characterization of alginate beads, such as calculating their volume, which can be correlated to their mechanical properties. Furthermore, Lin et al. [125] have observed the *in situ* shrinkage of alginate-based emulsion gel beads. It seems that the increase of Young′s modulus during gelation is combined with water loss and, consequently, shrinkage, leading to the formation of compact gelling structures. To conclude, the present section clarifies that alginate beads are produced mainly by extrusion-dripping methods, which can provide accurate visualizations of the formation process. Nevertheless, it is very challenging to characterize *in situ* the micro-structural conformations. The Table 1 presents cumulates the representative studies of Section 3 and Section 4 for the real-time recording of alginate gelation. 

**Table 1:** Recent representative methods for the real-time recording of alginate *in situ* gelation.

Technique	Experimental Details	Research Objectives	Year of Publication	Reference
Rheology	*In situ* release of cross-linker on alginate/gelatin mixture	Rheological investigation of gelation	2009	[63]
	Incubation of alginate in internal cross-linker on rheometer	*In situ* evaluation of gelation behavior		[66]
	LED bottom rheometric plate for UV cross-linking gelation	Real-time evaluation of mechanical properties	2011	[77]
	Rheological characterization upon direct mixing of reactants	Evaluation of gelation speed	2012	[116]
	*In situ* rheological characterization of fouled alginate membrane	Assessment of the parameters manifesting alginate fouling layer	2013	[107]
	Fluid hydrogel formation on rheometer	Controlling the shearing profile on gelation kinetics	2014	[68]
	UV irradiation on rheometer for photo-activated ionic gelation	Monitoring of the two-step gelation mechanism		[78]
	Rheological investigation upon ex situ mixing of reactants	Recording of gelation kinetics	2015	[69]
	Petri dish with filter paper and a dialysis membrane on the rheometer	Recording of alginate-Ca^2+^ kinetics	2016	[79]
	Visible light-curable alginate	Control of furfuryl alginate gel formation	2020	[76]
	Rheometric setup for injection of Ca^2+^ into alginate through micro-holes	Detailed recording of cross-linking gelation		[82]
	*In situ* micro-rheology of foulant membrane surface	Study on foulant mechanical response for effective cleaning	2021	[108]
	CO_2_ in alginate solution to initiate the gelation on the rheometer	Generation and characterization of hydrogel foams	2022	[86]
Microfluidics	Microfabrication of alginate hydrogels	Controlling of Ca^2+^ diffusion into alginate	2010	[99,100]
	Microfluidic flow-focusing device	Size and shape of the alginate-Ca^2+^ microparticles	2013	[117]
Visualization methods	Visualized extrusion-dripping method	Prediction models for alginate-Ca^2+^macrobeads	2009	[118]
	Visualization of gelation on a petri dish	Determination of sol–gel transition and gelling time	2011	[101]
	Photo correlation imaging during alginate gelation	Investigation of gelling kinetics formation	2013	[102]
	Flow cell between two microscope slides	Real-time studying of gel front	2016	[103,104]
	Microscopy on a drop-wise method	Microscopical characterization of alginate-Ca^2+^ beads	2017	[119]
	High-speed imaging system on drop impact gelation	Effect of drop kinetic energy on gelation	2018	[120,121]
	Custom setup for optical video microscopy	Measurement of alginate bead volume change over time	2021	[124]
Light-triggered methods	UV light in microchannels	Progress of UV-triggered gelation	2010	[98]
Capillary columns	Capillary formation of ionotropic alginate membranes	Propose equation for gelation kinetics	2013	[106]
Cantilever sensors	Dynamic-mode cantilever sensors on alginate gelation	Real-time monitoring of gelation	2020	[87,88]
Texture analyzer	Customized bead formation on a texture analyzer	*In situ* mechanical texture evaluation of alginate beads	2019	[123]
SAXS	Small-angle X-ray scattering oncompetitive ligand exchangegelation	Determination of the local structure of hydrogels	2019	[92]
Time-induced methods	FTIR, Uv-vis, and Raman spectroscopy on alginate–acrylamide gelation	*In situ* mapping of gel structure	2021	[95]

## 5. Conclusions and Outlook

The present review highlights the significance of real-time cross-linking alginate *in situ* gelation recording. Demonstrating such advanced experimental methods provides an assessment of the reaction progress and the main factors evolved in each stage. Customized rheometric setups and microscopical and structural time-resolved techniques have been developed over the last several years to control cross-linking gelation. Therefore, both mechanical and morphological properties can be recorded during the formation of alginate hydrogels and beads. This allows researchers to observe the process as it occurs and to investigate the effects of concentration and the physicochemical properties of reactants on the gelation evolved in an initial fast stage, followed by a slower evolution. Additionally, based on real-time experimental data, kinetic models are developed to describe and adequately predict the cross-linking alginate reaction. After extensive analysis of the research studies, it is observed that the fast external gelation, in which the gel point appears in time scales of seconds, can be quantified in terms of its mechanical properties and gel front position. Therefore, with the present review, an in-depth comprehension of alginate gelation is provided for the purpose of optimizing the conditions of the cross-linking reactions.

The prospects for *in situ* gelation are encouraging, since it provides many advantages compared to conventional gelation procedures. The transition of the liquid reactants to gelling structures on the characterization location, e.g., the rheometric bottom plate and microscopical cell, provides precise tuning of the mechanical and morphological properties over an extended time. The present review constitutes a valuable guide for identifying the appropriate technique to study the dynamic response, not only of alginate gelation, but also of other complex systems. The advanced setups can be utilized to study *in situ* sensitive biological hydrogels without the presence of an increased temperature which may cause a degradation of the material. Additionally, the *in situ* characterization of other cross-linking polymers, such as pectin and polyurethane, can be achieved. For the abovementioned reasons, developing methods to control the mechanical and structural characteristics simultaneously is deemed imperative. This will lead to the synthesis of smart hydrogels for state-of-the-art applications.

## Data Availability

Not applicable.
